# Determinant of under nutrition among under five children in Ambo town during covid 19 pandemic in 2020. A community-based cross-sectional study

**DOI:** 10.1186/s40795-023-00762-5

**Published:** 2023-09-15

**Authors:** Beshadu Bedada Feyisa, Getu Taresa Dabu

**Affiliations:** Department of Public Health, College of Medicine and Health Sciences, Ambo University, PO box 19, Ambo, Ethiopia

**Keywords:** Children, Stunting, Wasting, Underweight

## Abstract

**Background:**

About 8 to 44% of all child mortality in Africa is associated with undernutrition. To alleviate this problem, it is necessary to determine the magnitude and determinants of undernutrition during Covid 19 pandemic. However, there is scarce evidence in an urban setting like Ambo town. Therefore, this study assessed the magnitude and factors associated with undernutrition among under-five children in Ambo town, west Ethiopia.

**Methods and patients:**

A community-based cross-sectional study was conducted in Ambo town from March 01–30, 2020. The child and mother/caregiver socio-demographic characteristics, child illness and health care utilization, and child feeding practice-related data were collected using the standardized tool adopted from similar studies conducted in Ethiopia. Anthropometric measurements of the child were made using a calibrated scale. A systematic sampling technique was employed to select 363 mothers/caregivers of the child as a source of data. Nutritional status indices were generated using ENASMART software. After testing for collinearity, variables with a p-value < 0.25 in binary logistic regression were interred to backward multiple logistic regressions at a level of significance of p < 0.05.

**Results:**

A total of 363 participants were included in this study providing a 100% response rate and providing the following result. The mean (+ SD) age of children was 28.85 (+ 14.17) months and that of mothers/caregivers was 28.12 + 48 years. This study found 16.00%, 25.30%, and 19.00% of the study participants were underweight, wasted, and stunted respectively. Decisions making on major food purchases, who usually care for the child, the age at which the child starts complimentary food, late introduction of complementary food were positively associated with wasting. Diarrhea, birth weight, child age, age at which the child starts complimentary food, consumption of milk and milk product, and who usually care for the child were significantly associated with being underweight. Consumption of milk and milk products, household food security level, and birth weight were independent determinants of stunting.

**Conclusion:**

This study identified a high prevalence of undernutrition, especially wasting. Childbirth weight, age, diarrhea, feeding practice, household (HH) food security, Decision making on major food purchases, late introduction of complementary food were found to be the potential determinants of undernutrition. Thus there should be an effort to improve the nutritional status of children in the study area by focusing on these factors.

## Background

Globally, the prevalence of malnutrition among children under five years was unacceptably at a high level in 2019. About 144.0 million were stunted, 47 million were wasted and 38.3 million were overweight. About 40% of stunted and 27% of wasted children were in Africa. Being underweight is an alarming issue for low-income countries and can be ten times higher than in wealthier countries [1]. In Ethiopia, the results of the 2019 mini EDHS (Ethiopian Demographic and Health Survey) showed that 37%, 7%, and 21% of under 5 children were stunted, wasted, and underweight, respectively [2].

Undernutrition among under five years old children has both short-term and long-term impacts. These include social, economic, and health-related impacts [3–5]. In Africa, 8 to 44% of all child mortality is associated with undernutrition. Between 1 and 18% of all school, repetitions are associated with stunting. Stunted children achieve 0.2 to 3.6 years less in school education. Child mortality associated with undernutrition had reduced national workforces by 1 to 13.7%, and 40 to 67% of the working-age population suffered from stunting as children [6]. In Ethiopia, an estimated 55.5 billion ETB (Ethiopian Birr) was lost in the year 2009 as a result of child undernutrition. This is equivalent to 16.5% of GDP. This cost is related to the cost expended because of additional clinical episodes associated with undernutrition in children under five, increased child mortality, grade repetition rate, school dropout, and work hours lost, and 67% of adults in Ethiopia suffered from stunting as children [7].

Efforts to prevent the transmission of COVID-19 are disrupting food systems, upending health and nutrition services, devastating livelihoods, and threatening food security. UNICEF country offices reported a 30% decline in the overall coverage of services to improve nutrition outcomes for women and children in the early months of the pandemic [8]. Even without the added impact of the Covid 19, the world is not on track to meet Sustainable Development Goal 2 to end hunger and all forms of malnutrition [1]. In July 2020, the warning of the pandemic worsened the pre-existing crisis of malnutrition and tips an additional 6.7 million children over the edge to become wasted during its first year [9].

The economic impact of Covid 19 in developing countries is not the same as those of developed countries. Many adults in developing countries are self-employed and work in an informal sector with limited savings and access to safety nets [10]. In Ethiopia, measures to control the spread of the virus highly affect the urban residents, because their livelihoods are more likely to be in sectors that are more adversely affected by social distancing policies and travel bans including 14-day mandatory quarantine for international flights [11]. This can compromise diet quality, quantity, and diversity which increases the risk of undernutrition, especially among vulnerable groups in urban residents [8]. Currently, there is scarce literature on the nutritional status of under-five children during covid 19 in urban residences in Ethiopia. Hence this study aimed to determine nutritional status and its determinant among under-five children in Ambo town during covid 19 pandemic.

Apart from covid 19, the majority of previous studies conducted in Ethiopia focus on rural residents. Very few community-based studies were conducted on urban residents [12, 13]. These studies were conducted in the region where there is a high prevalence of undernutrition [12, 13]. The region where the previous studies were conducted is quite different from the region where the current study was conducted in terms of socio-economic status and culture including child feeding practice. In addition to this, these studies emphasized household, maternal and child characteristics, and economic variables [8–11, 14]. Furthermore, these studies overlooked more important variables like childbirth weight, maternal nutritional status, dietary diversity, and household food security [8, 13]. Thus, this study bridges the above-mentioned knowledge gap by assessing nutritional status and its determinant among under-five children in Ambo town. The results of this study will be used as baseline information for the researcher and for policymakers to make decisions and use available evidence-based interventions to improve the nutritional status of under-five children in the urban residence and in the context of covid 19.

## Study patients and methods

### Study Area, Design, and period

A community-based cross-sectional study was conducted from March 01–30, 2020, in Ambo town using a systematic sampling technique among children aged 6 to 59 months. Ambo town is the capital city of the West Shoa zone of the Oromia regional state which was found 144 km to the west of Addis Ababa. The town has a total population of 96,521off which 4869 are children of under-five years old. There are 02 public hospitals, 02 health centers, 32 private clinics, and 10 pharmacies. The livelihoods of the resident of the town majorly relied on the market and informal sectors. The town has six kebeles of which three kebeles were included in the study.

#### Study population

All sampled 6–59 months children residing in Ambo town were selected by systematic random sampling method.

### Exclusion criteria

All children with the following parameters were excluded from the study.

- Those who were critically ill at the time of the study.
- Those who did not volunteer to participate.
- Children whose family/caregivers were away from home during data collection for three consecutive visits were excluded from this study.

### Study variables, sample size, and sampling technique

#### Dependent variable

nutritional status measured as wasting, stunting, and underweight.

#### Independent variables

Seven categories of determinant factors were assessed as independent variables;

#### Socio-economic and demographic variables

Gender of the head of HHS, marital status, ethnicity, religion, family size, income, education, occupation, ownership of livestock and farmland, crop production, and home garden.

**Child characteristics**; Age, Sex, birth order, place of delivery, gestational age, types of birth, birth weight, and morbidity status.

**Child caring practices**; breastfeeding status, dietary diversity score (DDS), hygiene, health care seeking, and immunization.

**Maternal characteristics**; Age, number of children ever born, anti-natal care (ANC) visits, and autonomy in decision-making on major food purchases.

**Environmental health conditions**; safe water supply, sanitation, and housing condition.

The minimum sample size (n) required for this study was calculated using single population formula considering,

Zα/2 = is the standard normal score at confidence interval (CI) 95%=1.96.

p = proportion of stunting in Haramaya district 36.07% [15].

d = is the margin of sampling error tolerated 5% =0.05.

Since, the estimated population size is less than 10,000 (i,e there were only about 4869 children who are living in Ambo Town kebeles), a correction formula was used and a 10% non-response rate was also considered. Finally, 363 children were included in this study.

Three kebeles (Hora Ayetu, Sankale Farisi, and Ya’i Gada) were selected by lottery method and the final sample was proportionally allocated to the size of the participant in each selected kebeles. Finally, a systematic sampling technique without a sampling frame was used to select the study participant. The data collector makes the Kebeles office the center of the kebeles and goes to the four directions of the kebeles. They contact any household and count the first house where they got children of 6–59 months as one. They continue the same procedure until they reach the k value for each kebeles. The first household with children of 6–59 months to be included in the study was selected by the lottery method from the first household to k for each kebeles. Then they interview the study participant in the household in every kth value for each kebeles. K values vary for each kebeles. If there are two or more children of 6–59 months in the same household, one of them was selected by lottery method.

### Data Collection Tool, process, and Quality Management

A structured pretested questionnaire was used to collect the required data through face-to-face interview with the mother or primary caregiver of the child and anthropometric measurement was made for children and their mothers. The tool was adopted from similar studies conducted in a different part of Ethiopia including the Ethiopian demographic and health survey (EDHS) [2, 13, 15] and some possible modification was made to the tool after pretest, to fit the local context. The questionnaire was translated to Afan Oromo by one of the senior lecturers at Ambo University who is a fluent speaker of English and Afan Oromo for the field purpose and back-translated to English by another lecturer to check for consistency.

All anthropometric data were collected according to Food and Nutrition Technical Assistance( FANTA) anthropometric guide 2018 [16].

Weight was measured to the nearest 0.1 kg using a calibrated portable electronic digital scale (Seca). For children younger than 2 years old, the “tared weight” procedure was used. Children older than two years/were able to stand on a weight scale and mothers were measured with minimal clothes and without shoes. Weighing scales were calibrated with one-liter water regularly because its weight is known. The ace of scale indicator was checked against a zero reading for each measurement. Height/length was measured using a standardized measuring board to the nearest 0.1 cm. All anthropometric measurements were made two times and the average values were used for analysis. The child’s minimum dietary diversity score (MDDS) was measured using the 24-hour dietary recall method. For children, 6–23 months breastfeeding status was also considered the recall. The mother or primary caregiver of the child was requested to recall every food and beverage that was given to the child. Then the food group consumed by the child was coded to get MDDS.

Four public health graduating students were recruited and trained for four days on the tool, sampling technique, and obtaining informed verbal consent. The data collection was supervised by two field supervisors. The field supervisor and principal investigator checked the completeness, inconsistency, and inconvenience of data on the field and during summation.

### Statistical analysis

Anthropometric data were converted to nutritional status indices using ENASMART software and imported to Package for Social Science SPSS version 21 for analysis.

Before data analysis using SPSS version 21, all other data were cleaned, coded, and entered into the Epi data 3.1 version. Continuous variables were presented using mean with standard deviation. Frequencies and percentages were used to present categorical variables. After excluding variables with collinearity coefficients of > 0.8, variables with a p-value of < 0.25 on binary logistic regression were entered into backward multivariate logistic regression analysis with statistical significance at p-value < 0.05 to search for an independent determinant of all the indices of under-nutrition.

The household food insecurity level was measured with the Food Insecurity Experience Scale (FIES), a structured, standardized, and validated tool globally [17].

### Operational definition

#### Meet minimum dietary diversity score (MDDS)

those who fed at least 4 food groups among 7 food groups over the last 24 h before the interview [18] and for children aged 6–23 months, those who fed at least 5 food groups among 8 food groups including breast milk over the last 24 h before the interview [19].

#### Underweight

Refers to weight for age z score below the − 2 SD from the NCHS/WHO reference of the median of the standard curve [20].

#### Wasting

Nutritional deficient state of recent weight for height/length below-2SD from the NCHS/WHO median value [20].

#### Stunting

A child was defined as stunted if the height for age index was found to be below − 2 SD of the median of the standard curve [20].

#### Food secure

with raw scores = 0–3 to the questions about food insecurity-related experiences.

#### Moderate food insecurity

with raw scores = 4–6 questions about food insecurity-related experiences.

#### Severe food insecurity

with raw scores of 7–8 about food insecurity-related experiences [21].

#### Fully immunized

A child receiving all immunization recommended for his/her age according to recommended immunization for children in Ethiopia [22].

#### Partially immunized

A child that misses at least one of his/her immunization recommended for his/her age [22].

#### Not immunized

a child never took any immunization at all.

### Ethical consideration

Ethical clearance was obtained from Ambo University, College of Medicine and Health Science ethical review committee with the reference number AU/PGC1035/2020 on 20 February 2020. Confidentiality was kept and informed verbal consent was obtained from each mother/legal guardian of the children after explaining the purpose of the study. Using informed verbal consent was approved by the Ambo University College of Medicine and Health Science ethical review committee. This study was conducted following the ethical guidelines of the Helsinki Declaration.

## Results

### Socio-demographic characteristics of the study participants

A total of 363 participants were included in this study providing a 100% response rate. The mean (± SD) age of children was 28.85 (± 14.17) months and about 62.0% were male. Among all, 185 (51.0%) children were in the age category of 24–47 months and 259 (71.3%) of them had normal birth weight. Two hundred ninety-four (81.0%) children included in this study were living in male-headed household. About 92.8% of mothers of these children were living with the father of the children and about 49.6% and 41.0% of their father and mother respectively had an educational status of diploma and above. One hundred fifty-six (43.0%) of the household where these children living had severe food insecurity and 246 (67.8%) of both mothers and fathers of these children decide on major food purchases together. Two hundred twenty-five(62%) of the mother of these children were in the age group of 25–34 complete years and 98.3% of the household of the study participants had access to an improved water sources (Table 1).

**Table 1:** Socio-demographic characteristics of the study population

Socio-demographic characteristics of child’s family		frequency	percent
Child sex	male	225	62.0
	female	138	38.0
Child age (in completed month)	Mean ± SD 28.85 (± 14.17)		
	6–23	132	36.4
	24–47	185	51.0
	48–59	46	12.7
Birth weight	2.5–4.2	259	71.3
	< 2.5	30	8.3
	not weighted	74	20.4
Head of the household	male	294	81.0
	female	69	19.0
Current relationship of a mother with father of the child	mom live together	337	92.8
	mom lives alone with her child	26	7.2
Father educational status	diploma and above	180	49.6
	secondary education	73	20.1
	primary education	83	22.9
	No formal education	27	7.4
Mother educational status	diploma and above	149	41.0
	secondary education	68	18.7
	primary education	108	29.8
	No formal education	38	10.5
Mother occupation	housewife	135	37.2
	employed	197	54.3
	daily laborer	31	8.5
Maternal age	Mean ± SD = 28.12 ± 48		
	15–24	84	23.1
	25–34	225	62.0
	>= 35	54	14.9
Decision maker on major food purchase	both mother and father	246	67.8
	only one part of the family	117	32.2
Number of under-five children in household	1	295	81.3
	>= 2	68	18.7
	> 4	12	3.3
HH food security level	Food Secured	113	31.1
	Moderate food insecurity	94	25.9
	Sever food insecurity	156	43.0
Goat in household	yes	3	0.8
	no	360	99.2
Sheep in household	yes	28	7.7
	No	335	92.3
Chicken in household	yes	21	5.8
	no	342	94.2
Access to improved drinking water	yes	357	98.3
	no	6	1.7
Monthly income of the family	>= 4000	174	47.9
	< 4000	189	52.1

### Illness and health care utilization-related characteristics

Three hundred nine (85.1%) of the study participants were fully immunized and 214 (59.0%) got vitamin A supplementation in the last year before the interview. About 57.6% of the study participants experience at least one episode of illness in the last year and about 28.1% experiences diarrhea in the last two weeks. About 22.0% and 12.7% of the study children had fever and cough and fast breathing in the last two weeks respectively (Table 2).

**Table 2:** Illness and health care utilization-related characteristics

Health care utilization and health status of the child		Frequency	Percents
Immunization status for age	Fully immunized	309	85.1
	partially immunized	47	12.9
	not immunized	7	1.9
Vitamin A supplementation in the last year	yes	214	59.0
	no	149	41.0
Illness of the child last year	no	154	42.4
	yes	209	57.6
Diarrhea in the last two weak	no	261	71.9
	yes	102	28.1
fever in the last two weak	no	283	78.0
	yes	80	22.0
cough and fast breathing in the last two weak	no	317	87.3
	yes	46	12.7

### Caring and feeding practices of the study participant

About 76.9% of the study participants were usually cared for by their mother and 59.0% were breastfed appropriately. Two hundred eighty-four (78.2%) of the study participants started complementary food at six months and about 85.7% of them eat four or more food groups in the last 24 h before the interview (Table 3).

**Table 3:** Caring and feeding practices of the study participants

Caring and feeding practice		Frequency	Percent
Who usually feeds the child	mother	279	76.9
	Others*	84	23.1
ANC visit	yes	347	95.6
	no	16	4.4
Appropriate breastfeeding	yes	214	59.0
	No	149	41.0
Age at which the child starts complimentary food	start at six month	284	78.2
	start at < 6 month	67	18.5
	atart at > = 7 month	12	3.3
Dietary diversity score	>= 4 food groups	311	85.7
	< 4 food groups	52	14.3

_*_ Home maid, sibling, grandmother, father

### Food group consumed by the study participants

**Fig. 1:**
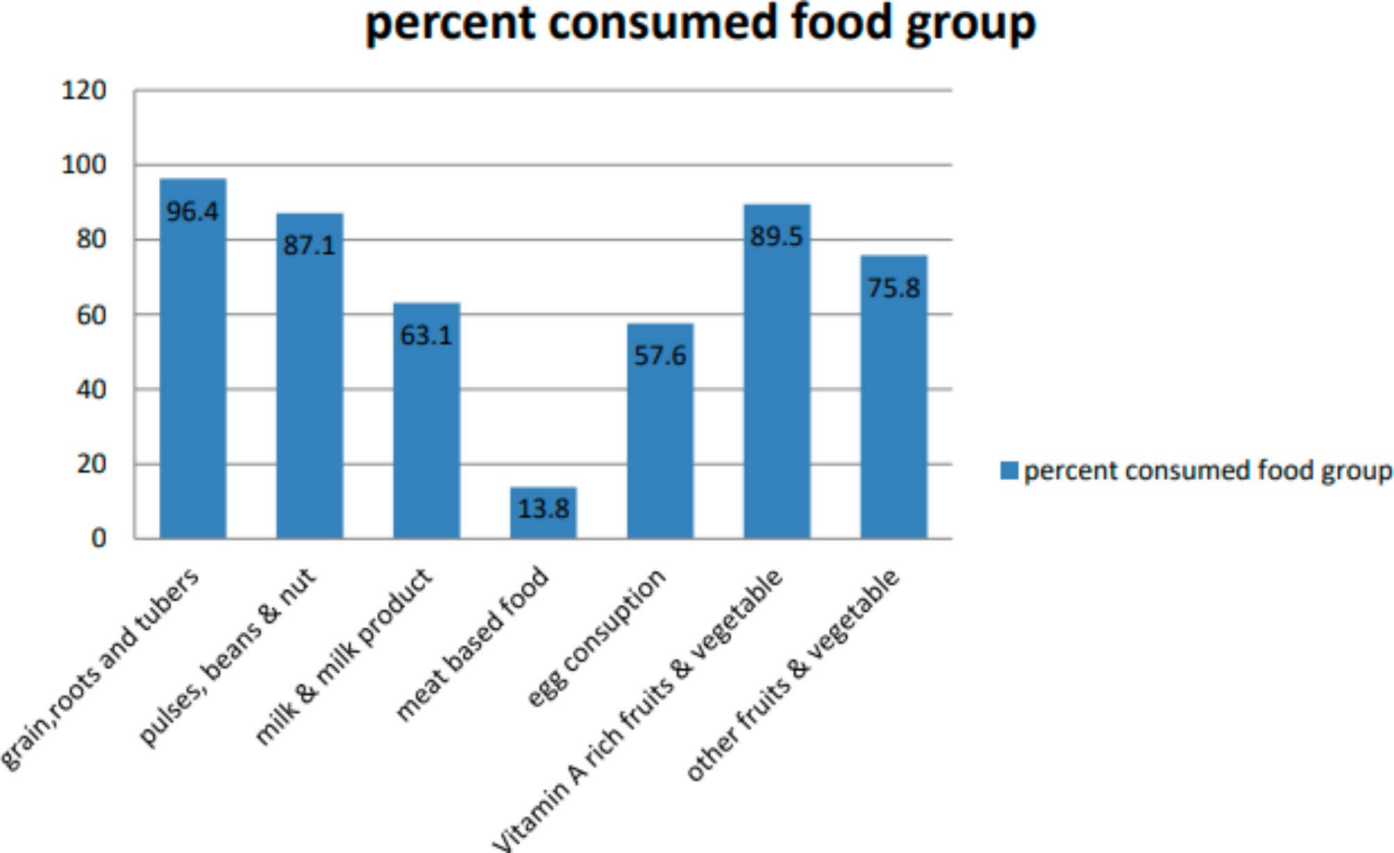
Food groups consumed by children

Most (96.4%) of the study participants consumed cereal-based food and few (13.8%) of them consumed meat-based food (Fig. 1).

### Nutritional status of the study participants

**Fig. 2:**
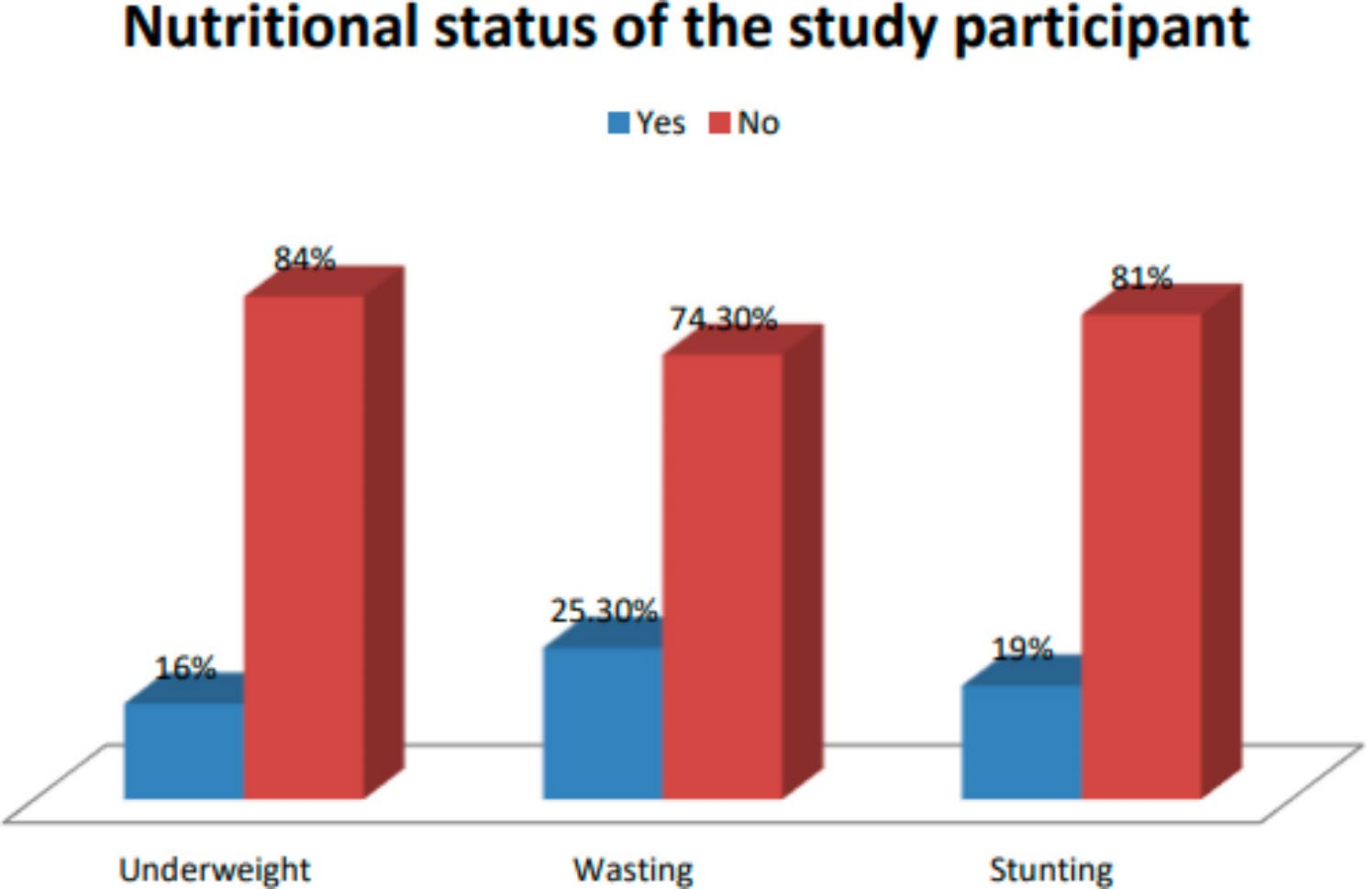
Nutritional status of children

About (16.00%), 25.30%, and 19.00% of the study participants were underweight, wasted, and stunted respectively (Fig. 2). About 23(39.66%) of underweight children were also stunted and 35(60.34%) of them were wasted. About 3(4.55%) of children who were stunted were also wasted.

### Determinants of wasting

Decision-making on major food purchases was an independent determinant of wasting among under-five children. Deciding on a major food purchases by only one member of the family increase the probability of wasting by two times (AOR = 2.512 at 95% C.I 1.426to 4.423, p-value < 0.0001). Children who were cared for by other people were less likely to waste by 60% (AOR = 0.407 at 95% CI 0.180 to 0.921) relative to children cared for by their mothers. Starting complementary food older than seven months increase chance of wasting by three times (AOR = 3.506 at CI 1.582 to 7.769) relative to those who start at six months (Table 4).

**Table 4:** Determinants of wasting

Variables	Categorical	Wasting status		AOR (95% CI)	COR (95% CI)	P-value
		No	Yes			
Decision maker on food purchase	Both mother & father	202 (82.1%)	44 (17.9%)	1	1	
	Only one part of family	69 (59.0%)	48 (41.0%)	3.237(1.461, 7.175)	2.512 (1.426, 4.423)	0.001
Fever in the last two weeks	No	200 (70.7%)	83 (29.3%)	1	1	
	Yes	71 (88.8%)	9 (11.2%)	0.414 (0.132, 1.300)	0.463 (0.209, 1.028)	0.058
Who usually cares for the child	Mother	195 (69.9%)	84 (30.1%)	1	1	
	Other people	76 (90.5%)	8 (9.5%)	0.452 (0.168, 1.214)	0.407 (0.180, 0.921)	0.031
ANC visit	Yes	259 (74.6%)	88 (25.4%)	1	1	
	No	12 (75.0%)	4 (25.0%)	0.297 (0.058, 1.515)	0.303 (0.077, 1.191)	0.089
Age at which complementary feeding started	At 6 month	206 (72.5%)	78 (27.5%)	1	1	
	< 6 month	56 (83.6%)	11 (16.4%)	1.458 (0.514, 4.135)	0.904 ( 0.500, 1.633)	0.737
	>= 7 month	9 (75.0%)	3 (25.0%)	0.869 (0.187, 7.289)	3.506 (1.582, 7.769)	0.002

### Determinants of Underweight

Diarrhea in the last two weak before data collection is the health condition of the child that was significantly associated with being underweight. Diarrhea increases the likelihood of being underweight by three times (AOR = 2.878 at 95% CI 1.206, 5.460, P = 0.014) relative to not having diarrhea. Birth weight is also the child’s nutritional status before birth that was significantly associated with being underweight. Being low birth weight increases the chance of being underweight during childhood by 7 times (AOR = 7.081, at 95% CI 2.650, 18.916, P < 0.001) compared to being normal birth weight. Older children more likely underweight by 8 times (AOR = 8.097, at 95% CI 3.090, 21.217, P < 0.001) compared to their counterpart. The age at which the child starts complimentary food is a feeding practice that was significantly associated with being underweight. Children who start complimentary food at older than seven months were 6 times underweight (AOR = 6.236 at 95% CI 1.376, 28.269, P = 0.018) compared to those who start at six months. Consumption of milk and milk products is another feeding practice that shows a significant association with being underweight. Children who do not consume milk and milk products were 3 times underweight (AOR = 2.878 at 95% CI 1.427, 5.804, P = 0.003) relative to those who consume milk and milk products 24 h before data collection. Children who were cared for by other people were less likely underweight by 80% (AOR = 0.197 at 95% CI 0.057, 0.680, P = 0.010) compared to those who were cared for by their mothers (Table 5).

**Table 5:** Determinants of underweight

Variables	Categorical	Underweight		COR (95% CI)	AOR (95% CI)	P value
		No	Yes			
Decision maker on food purchase	Both mother and father	215 (87.4%)	31(12.6%)	1	1	
	Only one part of family	90 (76.9%)	27 (23.1%)	1.062 (0.412, 2.737)	1.873 (0.922, 3.803)	0.083
Who usually cares for the child	Mother	225 (80.6%)	54 (19.4%)	1	1	
	Other people	80 (95.2%)	4 (4.8%)	0.500 (0.211, 1.185)	0.197 (0.057, 0.680)	0.010
Birth weight (g)	2500– 4200	231 (89.2%)	28 (10.8%)	1	1	
	< 2500	19 (63.3%)	11 (36.7%)	13.778 (3.309, 57.367)	7.081 (2.650, 18.916)	< 0.001
	not weighted	55 (74.3%)	19 (25.7%)	1.708 (0.563, 5.181)		0.175
Child age (in completed month)	6–23	120 (90.9%)	12 (9.1%)	1	1	
	24–47	159 (85.9%)	26(14.1%)	1.898 (0.587, 6.134)	2.098 (0.933, 4.716)	0.073
	48–59	26 (56.5%)	20 (43.3%)	26.323 (5.827, 118.899)	8.097 (3.090, 21.217)	< 0.001
Age at which complementary feeding started	At 6 month	236 (83.1%)	48(16.9%)	1	1	
	< 6 month	61 (91.0%)	6 (9.0%)	1.115 (0.182, 6.823)	1.089 (0,377, 3.145)	0.875
	>= 7 month	8 (66.7%)	4 (33.3%)	11.363 (1.078, 119.732)	6.236 (1.376, 28.269)	0.018

### Determinants of stunting

Consumption of milk and milk products was an independent determinant of stunting among dietary variables. Children who did not consume milk in the last 24 h before the interview were more likely stunted two times (AOR = 2.029 at CI 1.070, 3.665, P = 0.018) relative to those who does not consume milk and milk products. Children living in house hold with sever food insecurity has a chance of stunting by two times (AOR = 2.481 at 95% CI 1.198, 5.136, p = 0.014) compared their counterpart.

Birth weight is also an independent determinant of stunting. Being low birth weight increases the likelihood of stunting by three times (AOR = 3.185 at 95% CI 1.349, 7.518, P = **0.008**) relative to normal birth weight (Table 6).

**Table 6:** Determinants of stunting

Variables	Categorical	Stunting		COR (95% CI)	AOR (95% CI)	P-value
		No	Yes			
Number of under five children in HH	1	244 (82.7%)	51 (17.3%)	1	1	
	>= 2	50 (73.5%)	18 (26.5%)	2.499 (1.058, 5.905)	1.915 (0.980, 3.742)	0.057
Milk and milk product	Yes	195 (85.2)	34 (14.8)	1	1	
	no	99 (73.9)	35 (26.1)	2.065 (0.938, 4.544)	2.029(1.070, 30,529)	0.018
Maternal food security level	food secured	100 (88.5)	13 (11.5)	1	1	
	Moderate food insecurity	75 (79.8)	19 (20.2)	2.142 (0.807, 5.683)	1.811 (0.804, 4.079)	0.152
	Sever food insecurity	119 (76.3)	37 (20.2)	3.138 (1.27, 8.738)	2.481 (1.198, 5.136)	0.014
Having sheep	Yes			1	1	
	No			8.601 (0.850, 87.059)	6.127 (0.794, 47.293)	0.082
Birth weight (g)	2500– 4200	219 (84.6)	40 (15.4)	1	1	
	< 2500	18 (60.0)	12 (40.0)	2.799 (0.996, 7.864)	3.185 (1.349, 7.518)	0.008
	not weighted	57 (77.0)	17 (23.0)	1.717 (0.708, 4.165)	1.384 (0.691, 2.773)	0.369

## Discussion

This community-based cross-sectional study aimed at identifying the nutritional status and its determinant among under-five children during Covid 19 in an urban setting in West Shoa, western Ethiopia. Despite the national nutrition programs aimed to reduce the prevalence of undernutrition by 2020 [23], the current study identified that undernutrition among under-five children was high. This study noted decision-making on major food purchases, caring for the child, the age at which the child start complimentary food, diarrheal disease, birth weight, age of the child, consumption of milk and milk products, and household food security were associated with undernutrition among the study participant.

This study found a high prevalence of wasting and underweight as compared to the regional prevalence reported in mini EDHS 2019 [2] and studies conducted in Rwanda [24] and a high prevalence of wasting as compared to previous studies conducted in different parts of Ethiopia [12, 25, 26]. This high prevalence may be because of the small sample size as compared to the national wide survey. Another possible explanation for this discrepancy is the effect of covid − 19. The current study was conducted in an urban setting, where the impact of covid 19 worsened the food security of the household [27]. As the study conducted in 11 countries of Latin American countries reported a low level of diet quality among adult participants [28]. The low level of diet quality has an impact on the nutritional outcome of children, Covid-19 exacerbates all forms of malnutrition among the vulnerable group because of the deteriorating quality of their diet, interruption in nutrition and other essential services, and economic shock in low and middle-income courtiers [8]. A study done by Boutaina Zemrani and colleagues also reported that covid 19 significantly affects children’s nutrition and worsens undernutrition in developing countries [29].

This study found the prevalence of stunting (19%) lower than previous studies conducted in Ethiopia that identified the prevalence of stunting as 21–47.9% [12, 13, 25, 30]. This may be because of variations in the study area. Two of the study [12, 13] were conducted in the region where there is a high prevalence of stunting according to the mini EDHS 2019 report [2]. Other studies were conducted in rural and pastoralist communities where there is a high prevalence of undernutrition among under-five children [25, 31].

The current study found that child who’s both father and mother decides on major food purchase had good nutritional prognoses compared to those whose only one family member decides. This implies that both mother and father had power over major food purchases and they can fulfill the need of their child. Compared to this finding, a study conducted in rural communities in Ethiopia also found a significant association of power imbalance between the family and children under nutrition [32]. In line with this the finding of this study, a systematic review done in South Asia reported that women empowerment had a positive association with good child nutritional status [33].

In line with other studies conducted in different parts of Ethiopia [11, 13, 25, 34], this study found that diarrheal disease in the last two weak before the interview was found to be positively associated with being underweight. This may be because, infectious diseases play a major role in undernutrition as they result in increased needs and high energy expenditure, lower appetite, nutrient losses due to vomiting, poor digestion, malabsorption, and the utilization of nutrients and disruption of metabolic equilibrium [35].

In agreement with a study done in rural Ethiopia [36], this study identified that Children who start complimentary food at older than seven months were more likely underweight and wasted. At six-month infants triple their birth weight, they become active and their digestive system is also ready for food other than breast milk. At this age breast milk alone is no longer enough to meet their dietary need because of the increased demand for their growth and development [37]. There is evidence that the late introduction of complementary food increases the risk of undernutrition among under-five children [38].

Consumption of milk and milk products is another child-feeding practice that was found to be positively associated with stunting and being underweight. Milk contains high levels of energy, proteins, fat, and another micronutrient like calcium and the insulin-like growth factor-1 (IGF-1) that are of major relevance for children’s development and growth [39, 40]. A similar result was reported by studies that analyze the demographic and health survey (DHS) data of all low and middle-income countries [41]. Another study conducted in Tanzania found a significant association between milk consumption with arm circumference and stunting among female children of five years [42].

Similar to the findings of the evidence from the 2016 Ethiopian demographic and health survey [43], the current study identified that being low birth weight increases the likely hood of being underweight during childhood. Low birth weight is the fetal nutritional status that significantly affects the subsequent growth and development of the child and it is because of intrauterine growth restriction or prematurity [44]. It ends up in low growth with length, weight, head, and abdominal circumference that results in stunting and low weight due mainly to a lower proportion of visceral and fat tissue [45, 46].

Child age, being in the age group of 48–59 months increases the likely hood of being underweight by 8 times compared to being in 6–23 months. A similar finding was reported by another study in Ethiopia [24]. This may be because of insufficient dietary intake apart from their increased demand for their growth and development. Severe food insecurity increases the chance by two compared to food-secured households. This finding agrees with the findings of other studies done in Ethiopia [47]. This may be because food insecurity affects the dietary intake of the child.

This study accomplished its objective of assessing children’s nutritional status and its determinants in Ambo town. However, there are some limitations. First, it lacks information on some important confounding variables such as parasitic infection, HIV status, mother’s pre-pregnancy weight, and daily caloric intake which could cause problems in interpreting the results. Second, there may be a potential recall bias to collect data on the last 24-hour food consumption, birth weight, child’s history of illness, and breastfeeding practice.

However, these biases were reduced by using different technologies like looking at the birth certificate of the child for those who had it to look at birth weight and using a local calendar to help the respondent to memorize the child’s history of illness. Other variables related to covid 19 were also not measured because of the lack of tools and shortage of time to draft and validate tools during the pandemic.

## Conclusion

In conclusion, this study identified that high prevalence of undernutrition especially wasting. Decision-making on major food purchase, age at which the child starts complimentary food, diarrhea in the last two weak before data collection, birth weight, child age, consumption of milk and milk product, who usually care for the child, and household food security level were found to be the potential determinants of undernutrition (wasting, underweight and stunting).

There should be an effort on reducing child undernutrition. This could be true by reducing the incidence of diarrheal disease by increasing access to improved water, vaccination and sanitation, and hygiene. All stakeholders working on women’s affairs should work on empowering women in major household decision-making. The health office also advocates appropriate complementary feeding practices and the prevention of low birth weight. Stakeholders working on the economy of the community should work to improve the economic status of the community to ensure food security. Further study is needed to explore why children who were cared for by other people other than their mother were less likely undernourished. Further studies also need to identify the determinant of undernutrition relative covid 19.

## Data Availability

The datasets used and/or analyzed during the current study available from the corresponding author on reasonable request.
